# Microsatellite markers for the notothenioid fish *Lepidonotothen nudifrons* and two congeneric species

**DOI:** 10.1186/s13104-016-2039-x

**Published:** 2016-04-26

**Authors:** Chiara Papetti, Lars Harms, Jutta Jürgens, Tina Sandersfeld, Nils Koschnick, Heidrun Sigrid Windisch, Rainer Knust, Hans-Otto Pörtner, Magnus Lucassen

**Affiliations:** Alfred Wegener Institute Helmholtz Centre for Polar and Marine Research, Am Handelshafen 12, Bremerhaven, 27570 Germany; University of Bremen, BreMarE-Bremen Marine Ecology, Leobener Str. NW2, 28359 Bremen, Germany; Institute for Cell Biology and Zoology, Heinrich-Heine-University, Universitätsstrasse 1, Düsseldorf, 40225 Germany

**Keywords:** Antarctica, Gaudy notothen, *Lepidonotothen larseni*, *Lepidonotothen squamifrons*, Nototheniidae, Population differentiation, Southern ocean

## Abstract

**Background:**

Loss of genetic variability due to environmental changes, limitation of gene flow between pools of individuals or putative selective pressure at specific markers, were previously documented for Antarctic notothenioid fish species. However, so far no studies were performed for the Gaudy notothen *Lepidonotothen nudifrons*. Starting from a species-specific spleen transcriptome library, we aimed at isolating polymorphic microsatellites (Type I; i.e. derived from coding sequences) suitable to quantify the genetic variability in this species, and additionally to assess the population genetic structure and demography in nototheniids.

**Results:**

We selected 43,269 transcripts resulting from a MiSeq sequencer run, out of which we developed 19 primer pairs for sequences containing microsatellite repeats. Sixteen loci were successfully amplified in *L.nudifrons*. Eleven microsatellites were polymorphic and allele numbers per locus ranged from 2 to 17. In addition, we amplified loci identified from *L.* *nudifrons* in two other congeneric species (*L. squamifrons* and *L. larseni*). Thirteen loci were highly transferable to the two congeneric species. Differences in polymorphism among species were detected.

**Conclusions:**

Starting from a transcriptome of a non-model organism, we were able to identify promising polymorphic nuclear markers that are easily transferable to other closely related species. These markers can be a key instrument to monitor the genetic structure of the three *Lepidonotothen* species if genotyped in larger population samples. When compared with anonymous loci isolated in other notothenioids, i.e. Type II (isolated from genomic libraries), they offer the possibility to test how the effects of occurring environmental change influence the population genetic structure in each species and subsequently the composition of the entire ecosystem.

## Findings

### Research background

*Lepidonotothen nudifrons* (Lönnberg 1905), the Gaudy notothen [1] is a benthic and moderately active fish species belonging to the family Nototheniidae (red-blood species, [2]). *L. nudifrons* is especially abundant along the Islands of the Scotia Arc until the Bransfield Strait (Antarctic Peninsula) [1, 3]. This species plays an ecologically important role as prey to piscivorous fish species in this region [3], however, little is known about its genetic variability and the demography. Since the strongest local effects of climate change in polar regions were recorded for the Antarctic Peninsula [4], it is essential to investigate if *L.nudifrons* is experiencing any loss of genetic variability or if gene flow is limited among pools of individuals. Genetic differentiation among populations has already been demonstrated for another ecologically important notothenioid (*Pleuragramma antarctica*) in the same area [5].

These investigations require microsatellites, which have proven useful and reliable genetic markers in similar studies [5, 6]. In the past, the most common procedure to isolate microsatellites required the identification of repeat-containing sequences from libraries of genomic DNA enriched for microsatellite motives (Type II loci, [6–9]). In comparison to the traditional but labour-intensive and costly approach, currently these markers are also isolated from transcriptomic libraries obtained by next generation sequencing (NGS) that has proven to be more cost effective [7, 10]. However, markers obtained with this latter approach are gene-associated simple sequence repeats (SSR, Type I, functional loci, either called expressed sequence tags EST-linked markers [6, 9, 11]).

Due to their position inside or their flanking coding gene sequence and due to functional constraints, EST-linked SSRs are expected to have higher probability to be under selective pressure [6]. Gene-associated microsatellites have been isolated across many taxa in recent years [10 and references therein], however, only one study has reported isolation of microsatellites from a transcriptomic database in notothenioids [12].

The primary goal of this study is to expand the panel of available EST-linked loci in notothenioids for future studies on the evolution and demographic history of Antarctic fish. Moreover, we aim to isolate specific markers for *Lepidonotothen* spp. in order to compare their variability with anonymous Type II markers isolated in other notothenioid species.

We therefore mined microsatellite sequences from the publicly available spleen transcriptome of *L.nudifrons* [13] and screened several promising candidate loci for amplification and polymorphism. We were able to isolate sixteen species-specific SSR markers from assembled transcript sequences of *L. nudifrons.* Loci identified from *L.nudifrons* were also tested in two congeneric species: *L. squamifrons* and *L. larseni*.

## Methods

Gene-associated SSR markers were identified among 112,477 spleen transcripts of *L.nudifrons* [13]. The raw sequences were obtained from an Illumina MiSeq sequencer. Adapter clipping and quality trimming was performed using Trimmomatic v.0.32 [14] with following parameters: seed mismatch of 2, palindrome clip threshold of 30, simple clip threshold of 10, a minimum adapter length of 2, keep both reads parameter set to true, headcrop of 7, leading and trailing quality of 3, sliding window size of 4 with an average quality of 15 and a minimum sequence length of 50 bases. The subsequent *de novo* assembly was performed using the trinity genome-independent transcriptome assembler [release 17 July 2014, 15] with a minimum transcript length of 300 bases (for further details see [13]). Transcripts were screened using SciRoKo v. 3.4 [16]. Di-, tri-, tetra-, penta-, hexa-, hepta-, octa-, nona-, and deca-nucleotide repeat motifs were searched, setting the minimum repeat units to five, for all motif categories. Among transcripts containing microsatellites, we selected non-redundant SSRs with sufficiently large flanking sequences (>50 bp) on each side of the repeated units as ‘‘Potentially Amplifiable Loci’’ i.e. PAL [10, 17]. For subsequent analyses, we randomly selected 19 loci among all PAL. Primer pairs were designed with FastPCR v. 6.0 [18] to avoid primer dimers, self-annealing and hairpin formation when multiplexing loci during PCR. In addition, primers were designed to have very similar melting temperatures to avoid increased complexity of optimization protocol and to facilitate multiplexing.

Primer validation was carried out on genomic DNA extracted from 7 specimens of *L.* *nudifrons* collected in March/April 2012 near Elephant Island at 70–322 m depth during the RV Polarstern expedition ANT-XXVIII/4. The fish are not considered endangered at the sampling site. Sampling of the animals has been performed in accordance with the Antarctic treaty, and permission was given by the respective national authority (Umweltbundesamt: permit number I 3.5-94003-3/274). All treatments with live animals were in accordance with German law and approved by the competent national authority (Freie Hansestadt Bremen, Germany, permit number AZ: 522-27-11/02-00 (93)).

Total DNA was purified from 10 to 20 mg of muscle tissue following the standard protocol of the DNeasy Blood and Tissue Kit (Qiagen, Germany). Quality and quantity of DNA extractions were assessed using a NanoDrop™ 2000c spectrophotometer (Thermo Fisher Scientific, USA) before samples were stored at −20 °C.

Initially, PCR primer pairs were tested as single-locus PCR in 20 μl volume, containing 1X reaction buffer (5 Prime, Hamburg, Germany), 70 μM dNTPs, 0.25 μM of each primer, 1 unit Taq polymerase (5 units/μl, 5 Prime, Hamburg, Germany) and ~30 ng of genomic DNA. PCR conditions were: initial denaturation at 94 °C for 1 min, followed by 30 cycles of 94 °C for 30 s (denaturation), 54 °C for 40 s (annealing), 72 °C for 40 s (extension) and a final single extension step at 72 °C for 5 min. Electrophoresis was carried out at 100 V on 1.5 % agarose gels containing GelRed Nucleic Acid Gel Stain (Biotium, Hayward, USA) for a preliminary qualitative polymorphism detection. Only loci that provided a clear PCR product were retained (for details, see Results section) and their polymorphism was verified on an Applied Biosystems 3130 XL automated sequencer (Life Technologies, USA, ROX500 as size standard) using a larger sample of 21 *L.* *nudifrons* individuals (collected in March/April 2012 near Elephant Island, Research Vessel RV Polarstern, expedition ANT-XXVIII/4, DNA extraction as described above). To this purpose, forward primers were labelled with fluorescent dyes FAM, HEX, and TAMRA (Applied Biosystems, USA) and loci were combined in multiplex PCRs designed with Multiplex Manager v. 1.2 [19]. Amplification reactions were optimised in terms of volumes and concentrations of reagents to reduce the total genotyping cost. Amplifications were carried out in 10 μl reaction volume, using the Multiplex PCR kit (Qiagen, Germany) in accordance with manufacturer’s instructions. PCR conditions were: initial denaturation at 95 °C for 15 min, followed by 30 cycles of 94 °C for 30 s (denaturation), 57 °C for 90 s (annealing), 72 °C for 60 s (extension) and a final single extension step at 60 °C for 30 min.

Specimens of *L.* *squamifrons* (n = 20) and *L.larseni* (n = 18) were tested to assess loci variability in congeneric species (DNA extraction and amplification conditions as described above). Individuals of these two species were collected around Elephant Island in March/April 2012 (RV Polarstern expedition ANT-XXVIII/4).

Allele sizes were assigned using GeneMarker v. 2.6.3 (Soft-Genetics, Pennsylvania). Binning was automated with Flexibin v. 2 [20]. All input files for statistical analysis were produced with Create v. 1.37 [21]. All standard and basic statistics were produced with DiveRsity v. 1.9 [22]. The estimation of departure from Hardy–Weinberg equilibrium (HWE) was obtained with Genepop (online version, exact test) [23]. The presence of null alleles was assessed using the program ML-NullFreq [24]. Genotypic disequilibrium for pairs of loci (Fishers' exact test) was tested with Genepop (online version) [23]. Correction for multiple testing (HWE and genotypic disequilibrium) was accomplished using the standard Bonferroni technique [25, 26].

To annotate the transcripts corresponding to the final panel of markers, homology searches were performed using Blastx [27] against the UniProtKB/Swiss-Prot database with an e-value cut-off of 10^−9^.

## Results

We identified 43,269 transcripts containing SSRs with 2–10 repeat units out of 128 Mb transcripts of a spleen transcriptome (Table 1). This result indicated an average amount of one SSR per 2.9 Kb. The number of transcripts containing SSR represented approximately 38.46 % of the total sequenced transcriptome. The largest proportion of SSRs (82.50 %) consisted of di-nucleotide repeats. SSRs with nucleotide repeat of higher complexity (e.g. tri- to deca-nucleotide repeat motifs) were present in progressively smaller numbers (Table 1). A list of all the transcripts containing SSR motifs can be provided upon request. 484 transcripts resulted to be suitable for primer design (PAL), and 19 of these sequences (containing a di-nucleotide repeat motif) were randomly chosen for primer design. Of these 19 loci, two did not provide any amplified fragment, even after attempting to optimize annealing temperatures. One locus showed a longer fragment than expected based on the original sequence (length determined by agarose gel-sizing), possibly suggesting the presence of an intron and therefore making allele sizes unpredictable [11]. These three markers were discarded from subsequent analyses. The remaining 16 markers showed a clearly defined amplified band on agarose gel and could be easily genotyped with an automatic sequencer in three multiplex PCRs containing eight, six, and two loci. The final panel of markers (Tables 2 and 3) consists of five monomorphic and eleven polymorphic loci for *L.* *nudifrons* with no missing genotypes. The allele number per locus ranged from 2 to 17 (Table 3), with an average value of 5.1 (±4.4 SD). Mean observed (H_o_) and expected heterozygosities (H_e_) were 0.43 (±0.34 SD) and 0.43 (±0.31 SD). Ten loci out of eleven were in HWE after correction for multiple testing (nominal significance level α = 0.05, Table 3). Hardy–Weinberg disequilibrium was detected for locus Ln_42016, due to excess of homozygosity. This result could be due to several reasons, such as single locus stochasticity due to small sample size (n = 21) or occurrence of a non-amplified allele. The presence of a null allele was suggested by ML-NullFreq [24] at this locus with a frequency of 4.9 %. Loci with null alleles might have impacts on estimates of population differentiation [28] and are generally not recommended for use in population genetic inference. However, it has been shown that the influence of null alleles in studies of population genetics might be marginal compared to other factors such as the number of loci and strength of population differentiation [29]. Departure from HWE due to excess of homozygosity could also be caused by other factors either to scoring errors or amplification artefacts. Departure from HWE could be generated by the pressure of actually occurring evolutionary forces (e.g. selection, local adaptation) or admixture of genetically distinct populations (i.e. Wahlund effect [30]). This can affect loci at different magnitude with some triggers being locus-specific and others being sample-specific [31]. In some circumstances, the usual approach of consistently removing loci in Hardy–Weinberg disequilibrium may be too conservative leading to the exclusion of ecologically informative markers [31, 32].

**Table 1:** Summary of Simple Sequence Repeats (SSR) found in the transcriptome of *Lepidonotothen nudifrons* [13]

Type of repeat (length of motif in number of nucleotides)	No. of transcripts
SSR (all motifs)	*43,269*
SSR (2)	35,697
SSR (3)	6905
SSR (4)	549
SSR (5)	32
SSR (6)	23
SSR (7)	5
SSR (8)	15
SSR (9)	26
SSR (10)	17
PAL SSR with ≥ 8 repeats (all motifs)	*484*
PAL SSR (2)	478
PAL SSR (3)	5
PAL SSR (4)	1

SSR were searched among 112,477 transcripts. PAL, Potentially Amplifiable Loci [10, 17]

**Table 2:** Characteristics of 16 SSR loci in *Lepidonotothen nudifrons* and two additional congeneric species

Locus name	Accession #	Blastx Top Hit	Repeat content	Primers (5′–3′)	Fluorescent label	Multiplex mix	Ln	Ls	Ll
Ln_22268	HACN01006381	–	(GA)_9_	F: TGGATGGTTTTTCGTTTGACCAA R: TGAAGAGCTTTGGAGCAACAA	TAMRA	1	M	M	M
Ln_22517	HACN01006568	–	(AT)_9_	F: TGATTCATCCTCTGGGTACCAT R: AGCTCGTAGCTATTCCAGCA	HEX	2	P	M	–
Ln_23194	HACN01007070	–	(TA)_9_	F: TCCGGAGGCTGCGTTCAGAG R: CTGCTTGAGGACCGGCTCAG	HEX	1	M	P	P
Ln_32298	HACN01014077	Dolichol-phosphate mannosyltransferase subunit 3 (Q3ZC71)	(AC)_9_	F: AAAGTAGTCCGTGCATCCCTT R: GTCTTACGACCTGTTGGAGC	TAMRA	2	M	M	M
Ln_34878	HACN01015998	–	(AG)_10_	F: GTTGCAGTCACGTTGAAAGC R: ACGAATCCAAACCTTGCAACAT	FAM	3	P	–	–
Ln_35217	HACN01016251	–	(CT)_9_	F: CCACTACACAGCTGAAATGGT R: GGCATGATTGGAGCACTTCC	HEX	1	P	P	P
Ln_36100	HACN01016925	–	(AG)_9_	F: TAAAGCTCCAGCCGATACTGG R: TGGGAATTGAACAGATGTCACC	TAMRA	1	P	M	P
Ln_36156	HACN01016972	–	(GT)_9_	F: GTCTGCTCATGTCCAACACAT R: AGGTGTTTCTGTAAACCCTCG	FAM	2	P	P	P
Ln_40551	HACN01020461	–	(TA)_9_	F: TAACTGATGCATGCCCAGGAACTT R: GCTTCCTAAAAGCTCCACAGAT	FAM	1	P	P	P
Ln_41246	HACN01021014	–	(AT)_9_	F: GCTTAAATCCAACCATAGACGCA R: CTCATCAGGACTTCACGTGC	FAM	2	M	–	–
Ln_41281	HACN01021039	Uncharacterized protein C1orf21 homolog (Q8K207)	(GT)_9_	F: TGTGTTTTTGGACGTATGGCA R: GCTTCCATTTTGGGAGTACCT	HEX	1	P	P	P
Ln_42016	HACN01021779	–	(GT)_9_	F: ACATCACTTGCAAATAGGCCGGTG R: CCGTCATGCACCTGGGAGTT	HEX	3	P	P	P
Ln_42233	HACN01021981	Serine/threonine-protein kinase N2 (A7MBL8)	(AT)_9_	F: ATTCCTGACTGCCAAAGACG R: TGTTGACTTGCAGACTGAACG	FAM	1	P	M	P
Ln_42701	HACN01022446	Hepatic leukemia factor (Q8BW74)	(AT)_9_	F: TGGACTTCACAAGCGTGGCT R: ATTGAAAGTGTGCATAGTCCGT	FAM	1	M	M	M
Ln_45257	HACN01024934	–	(TG)_9_	F: AAGACCAGGGCGAGTCTGAC R: CTCTAACCAGAACACCGCCT	HEX	2	P	P	P
Ln_45589	HACN01025236	–	(AG)_9_	F: CGTGTCTAGGAGCTACAGCA R: CCAGGTTGGTGGCATCTCTT	TAMRA	2	P	P	P
						Total	11	8	10

The table reports: the name of each locus (Ln = *Lepidonotothen nudifrons* and the transcript number), Accession number of the *de novo* assembled transcripts of *L.* *nudifrons* (European Nucleotide Archive -ENA- project number PRJEB8919) [13], the putatively annotated loci (Blastx Top Hit, e-value < 10^−9^), the repeat content, the forward (F) and reverse (R) primer sequences, the fluorescent label, the multiplex pool (mix). Variability, expressed in terms (P) polymorphic, (M) monomorphic or (-) unsuccessful locus, was assessed on 21 *L.* *nudifrons* (Ln), 20 *L. squamifrons* (Ls) and 18 *L. larseni* (Ll) individuals. Total number of polymorphic loci is reported in the last row

**Table 3:** Summary statistics of 16 SSR loci genotyped in *Lepidonotothen nudifrons, L. squamifrons* and *L. larseni*

Locus name	*Lepidonotothen nudifrons* (21)					*Lepidonotothen squamifrons* (20)					*Lepidonotothen larseni* (18)				
	Size range (bp)	Na	H_o_	H_e_	pHWE	Size range (bp)	Na	H_o_	H_e_	pHWE	Size range (bp)	Na	H_o_	H_e_	pHWE
Ln_22268	108	1	0	0	–	108	1	0	0	–	108	1	0	0	–
Ln_22517	251–263	4	0.29	0.26	1	245	1	0	0	–	–	–	–	–	–
Ln_23194	92	1	0	0	–	81–92	2	0.05	0.05	1	91–94	2	0.22	0.28	0.3899
Ln_32298	275	1	0	0	–	275	1	0	0	–	275	1	0	0	–
Ln_34878	126–138	7	0.86	0.8	0.0052	–	–	–	–	–		–	–	–	–
Ln_35217	242–246	3	0.33	0.42	0.1606	237–256	6	0.7	0.63	1	240–252	6	0.83	0.68	0.8929
Ln_36100	258–264	2	0.1	0.09	1	263	1	0	0	–	240–263	3	0.22	0.42	0.0326
Ln_36156	186–244	17	1	0.92	0.9627	191–241	14	0.8	0.86	0.1197	184–248	16	0.89	0.92	0.6330
Ln_40551	291–329	6	0.52	0.6	0.4682	272–294	2	0.5	0.38	0.2770	287–314	10	0.17	0.87	*P* *<* *0.0001*
Ln_41246	89	1	0	0	–	–	–	–	–	–	–	–	–	–	–
Ln_41281	314–320	2	0.1	0.09	1	314–320	2	0.35	0.44	0.3492	314–320	3	0.22	0.2	1
Ln_42016	127–139	7	0.62	0.73	*P* *<* *0.0001*	135–139	2	0	0.45	*P* *<* *0.0001*	135–139	3	0.11	0.32	*0.0029*
Ln_42233	212–242	2	0.1	0.09	1	214	1	0	0	–	212–242	3	0.22	0.29	0.0611
Ln_42701	107	1	0	0	–	107	1	0	0	–	107	1	0	0	–
Ln_45257	98–100	2	0.05	0.13	0.0731	78–92	3	0.05	0.43	*P* *<* *0.0001*	78–101	3	0.17	0.25	0.0392
Ln_45589	132–146	4	0.76	0.64	0.1007	129–171	8	0.7	0.63	1	131–137	4	0.61	0.55	0.9367

For each species (sample size in brackets), the table shows the size range of amplified fragments in bp, the number of alleles (Na) detected, the observed (H_o_) and expected (H_e_) heterozygosities and the Hardy–Weinberg equilibrium probability (pHWE). Significant p-values, after correction for multiple testing using the standard Bonferroni technique [25, 26] are in italics (nominal significance level α = 0.05)

Although our loci are located in transcribed sequences, only three loci could be putatively annotated through similarity search (Blastx, Table 2) [27]. A fourth marker resulted in a similarity to an “uncharacterized protein C1orf21 homolog” (Table 2). These transcripts are likely portions of protein-coding genes and future studies should aim at combining information about allelic frequencies, gene expression and function. All polymorphic loci were in linkage equilibrium after correction for multiple testing (nominal significance level α = 0.01, data not shown).

In the congeneric species, *L. squamifrons* and *L. larseni*, two loci failed to amplify consistently despite attempts to optimize the PCR conditions. Locus Ln_22517 was successfully amplified in *L. squamifrons* (monomorphic), but it did not provide a consistent amplification and genotyping result for *L. larseni*. The remaining 13 markers were successfully genotyped (Table 3). Eight loci were polymorphic in *L. squamifrons*, while in *L. larseni* we scored multiple alleles in ten loci (Tables 2 and 3). In particular, Ln_23194, monomorphic in *L. nudifrons*, turned out to be polymorphic in *L. squamifrons* and *L. larseni* (Tables 2 and 3). We obtained a panel of loci that worked for all three species at the same PCR conditions, which facilitates the rapid implementation and application at large sample size scale.

Our results confirm that polymorphic SSR markers can be effectively isolated from transcriptomes of non-model organisms [12, 33]. For *Lepidonotothen* spp. these markers are also easily transferable among different, but phylogenetically closely related species.

Additional tests should be implemented to verify whether these loci could be considered candidates for being influenced by selection. An effective method to verify this, is to compare F_ST_ values to search for loci showing a significantly high level of genetic differentiation between pairs of species as applied for the *Chionodraco* genus [6]. Agostini et al. [6] indicated that out of 21 microsatellites two Type I markers and one Type II locus were putatively under selection.

Moreover, we aim to use our microsatellites in conjunction with other markers such as mitochondrial and nuclear sequences and on a larger sample size. This would effectively help monitoring the genetic structure of the three *Lepidonotothen* species, profiling demographic events occurred in the past, and identifying signatures of local adaptation in genetically different populations. To understand the significance of these events and the climate challenges that marine organisms in polar regions are facing, is essential to model and project future population viability for species management and conservation [34].

## Abbreviations

ENA: European nucleotide archive
EST: expressed sequence tags
H_e_: expected heterozygosity
H_o_: observed heterozygosity
HWE: Hardy–Weinberg equilibrium
NGS: next generation sequencing
PAL: potentially amplifiable loci
RV: research vessel
SSR: simple sequence repeats
